# Unilateral Congenital Pulmonary Hypoplasia: A Rare Cause of Severe Respiratory Distress in a Three‐Weeks‐Old Neonate

**DOI:** 10.1002/ccr3.71383

**Published:** 2025-11-02

**Authors:** Bernadette Pedun, Ivaan Pitua, Samuel Bugeza, Anthony Oriekot, Felix Bongomin

**Affiliations:** ^1^ Uganda Cancer Institute Kampala Uganda; ^2^ School of Medicine, College of Health Sciences Makerere University Kampala Uganda; ^3^ Department of Radiology and Radiotherapy, School of Medicine, College of Health Sciences Makerere University Kampala Uganda; ^4^ Mulago Specialized Women and Neonatal Hospital Kampala Uganda; ^5^ Department of Internal Medicine Gulu Regional Referral Hospital Gulu Uganda; ^6^ Department of Medical Microbiology and Immunology Gulu University Gulu Uganda

**Keywords:** case report, chest computed tomography angiography, congenital pulmonary hypoplasia, lung hypoplasia

## Abstract

This case of a unilateral pulmonary hypoplasia in a newborn with severe respiratory distress demonstrates the importance of early chest computed tomography angiography in diagnosing congenital lung malformations. Rapid, accurate diagnosis enables appropriate neonatal intensive care unit support and informs prognosis, highlighting the value of imaging in neonatal respiratory emergencies.

## 1 Introduction

Congenital pulmonary hypoplasia is a rare and serious condition characterized by incomplete lung development, affecting one or both lungs in the fetus [1, 2, 3]. This lung hypoplasia occurs in approximately 9–11 per 10,000 live births, with a high mortality rate of 71%–95% [3, 4] and may arise independently or alongside other anomalies [5].

In neonates, the lungs are essential for gas exchange to transition from intrauterine life. Hypoplasia disrupts this, leading to respiratory distress and potentially fatal outcomes within the first 48 h. Causes are not fully understood and are thought to be idiopathic or multifactorial, involving genetic factors or intrauterine issues like oligohydramnios or chromosomal anomalies [3, 6, 7].

Antenatal ultrasound may identify hypoplasia by revealing thoracic size discrepancies and structural anomalies. Diagnosing pulmonary hypoplasia postnatally, especially in a three‐week‐old neonate, requires thorough evaluation and imaging. Management demands a multidisciplinary approach, including immediate ventilatory support, with surgical interventions considered as appropriate. We herein report a case of congenital pulmonary hypoplasia in a term neonate from Uganda.

## 2 Case History/Examination

We present a case of a 3‐week‐old male neonate, weighing 3.0 kg, born at term via an uncomplicated vaginal delivery to a 26‐year‐old primigravida mother. The prenatal period was uneventful, and there were no reported maternal infections or exposures nor a recall of any family history of congenital lung or respiratory disorders. Prenatal ultrasound examinations were non‐informative; this was a verbal report from the mother that her antenatal scan did not reveal any fetal anatomical anomalies. It was not ascertained at what gestational age the scans were done, who performed the scans, what they were interested in finding, and whether an anatomy scan was performed at the right gestational age.

Mother reports that he did not cry immediately after birth and shortly after developed breathing problems that required immediate resuscitation and was transferred to the neonatal intensive care unit (NICU) for further evaluation and management. While in the NICU, the baby had been on ventilatory support to assist with his breathing and unspecified antibiotics. Despite the intervention, his respiratory distress persisted, and oxygen saturation levels remained suboptimal.

The clinical exam findings were extracted from the patient chart while still under the care of the NICU team, which consisted of paediatricians, intensivists, surgeons, and neonatal nurses. The exam findings pointed towards a pulmonary cause, such as respiratory distress syndrome, meconium aspiration syndrome, congenital pneumonia, and pulmonary hypoplasia.

Complete Blood Count results showed normal white blood cell count for age and no signs of infection. Arterial Blood Gas Analysis demonstrated significant hypoxemia and respiratory acidosis. Following the patient's clinical presentation, further radiological evaluation with a Computed Tomography Angiogram (CTA) of the chest was conducted.

**FIGURE 1:**
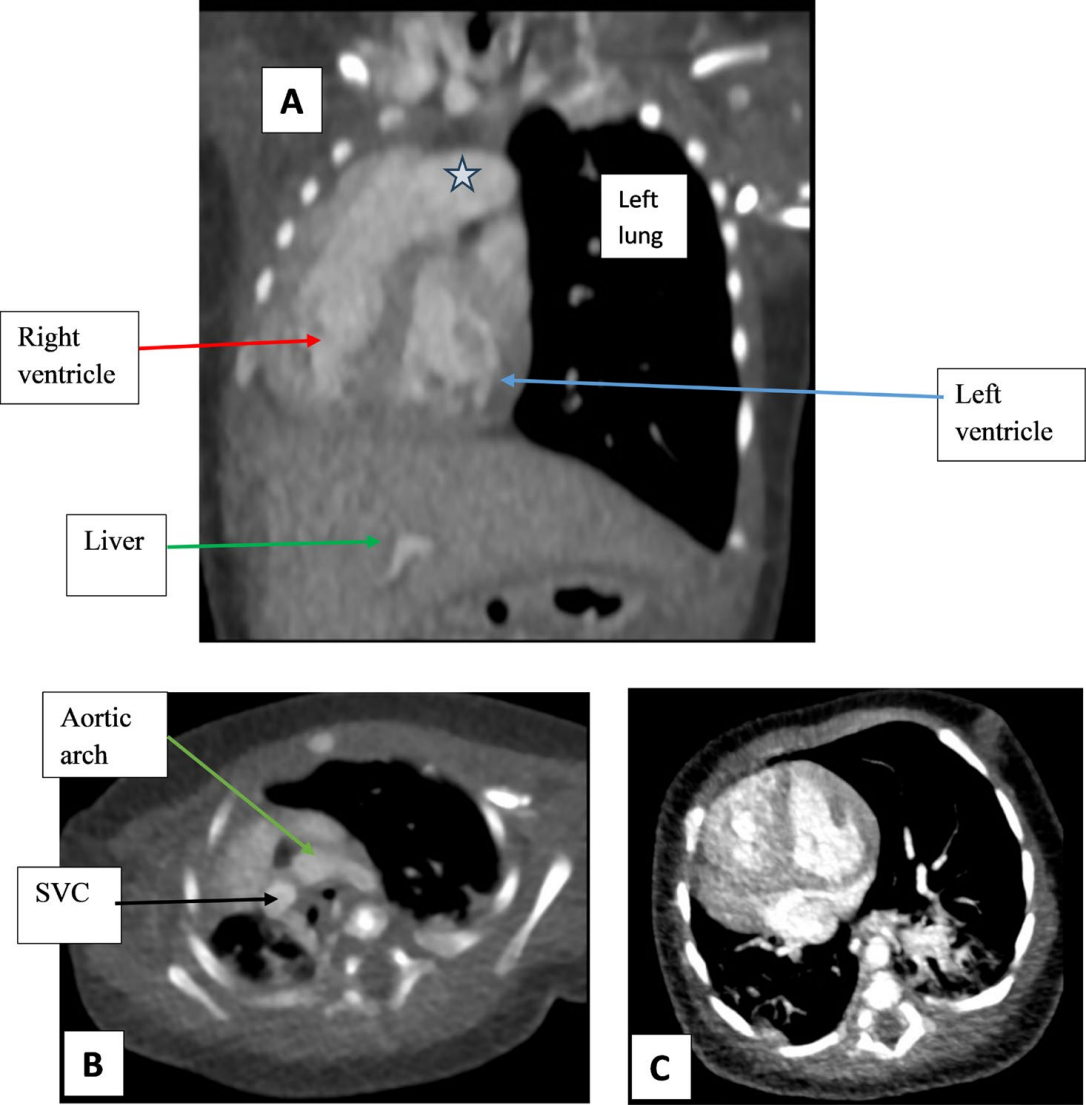
(A, B C) Post I.V contrast CT images of the chest, (A) Coronal reformat, (B, C) Axial cuts, soft tissue window. There is severe deficiency of the right lung with mediastinal shift to the ipsilateral side, a globally enlarged heart with a notably dilated main pulmonary artery (star in image A) but the levocardia is maintained with a left‐sided aortic arch noted and normal mono SVC. Image C shows the mediastinal shift and reduced right lung volume. There was no intracardiac shunt noted. No diaphragmatic defects or pleural effusions are noted.

**FIGURE 2:**
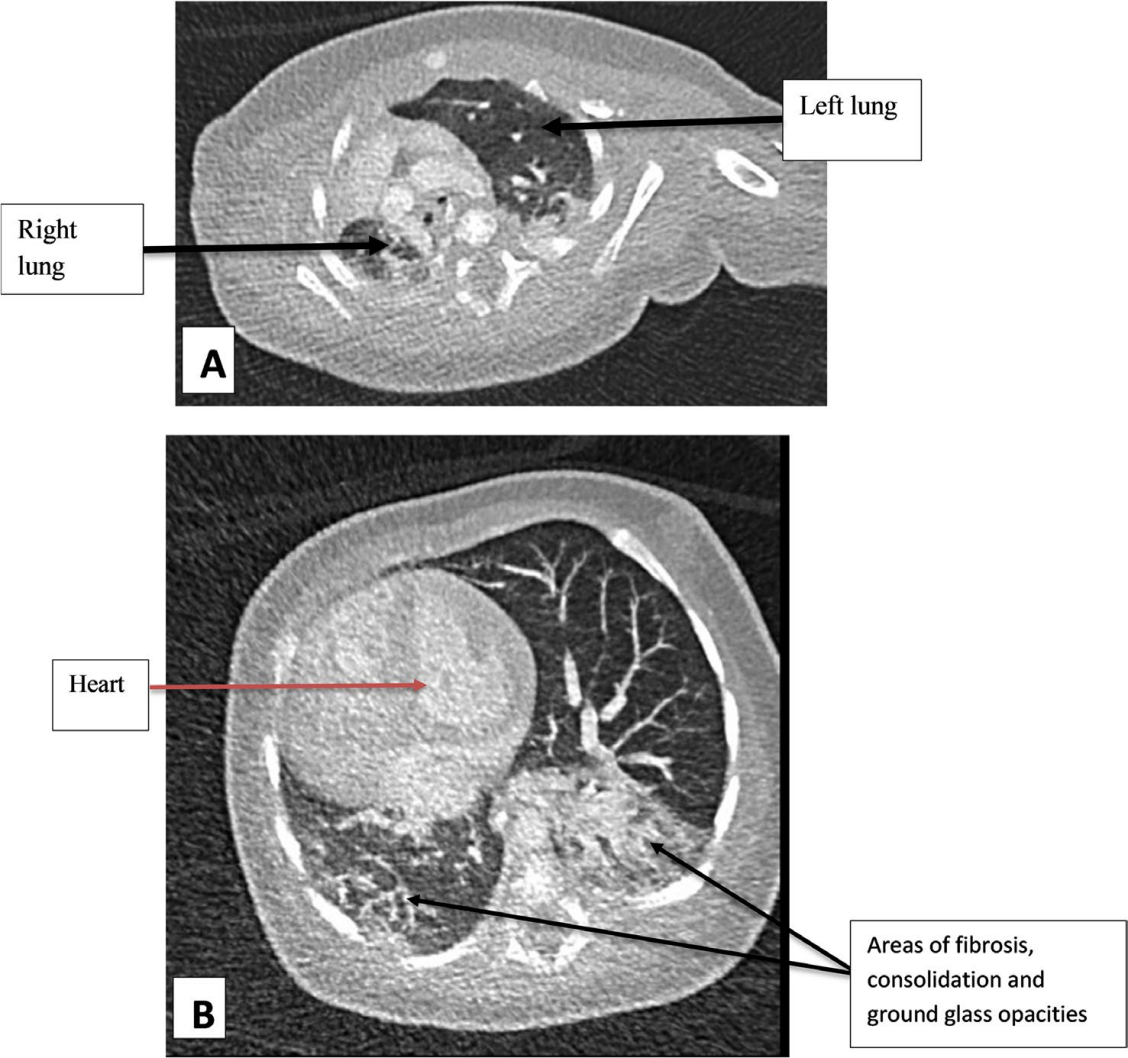
(A, B) Chest CT images in axial slices, lung window at the level of the lung apices and the ventricles respectively. The right lung is hypoplastic and the left lung is hyperinflated, crossing the midline. There are ground‐glass opacities, consolidations and fibrosis bilaterally, the right side worse than the left.

The CTA findings (Figure 1) demonstrated significant right lung hypoplasia, evidenced by severe deficiency of the right lung and an ipsilateral mediastinal shift. Additionally, the right lung exhibited extensive fibrosis, with the left lung compensatorily hyperinflated and crossing the midline (Figure 2). There was also a globally enlarged heart with a prominently dilated main pulmonary artery (Figure 1).

The CTA images revealed no evidence of an intracardiac shunt, diaphragmatic defects, or pleural effusions, and the levocardia remained intact, with the aortic arch positioned on the left and a normal single superior vena cava (SVC) observed (Figure 1). The axial slices at the level of the lung apices and ventricles provided further insights into the left lung's hyperinflation and right lung hypoplasia, showing ground‐glass opacities, consolidation and fibrosis across multiple segments bilaterally, the right worse than the left (Figure 2).

**FIGURE 3:**
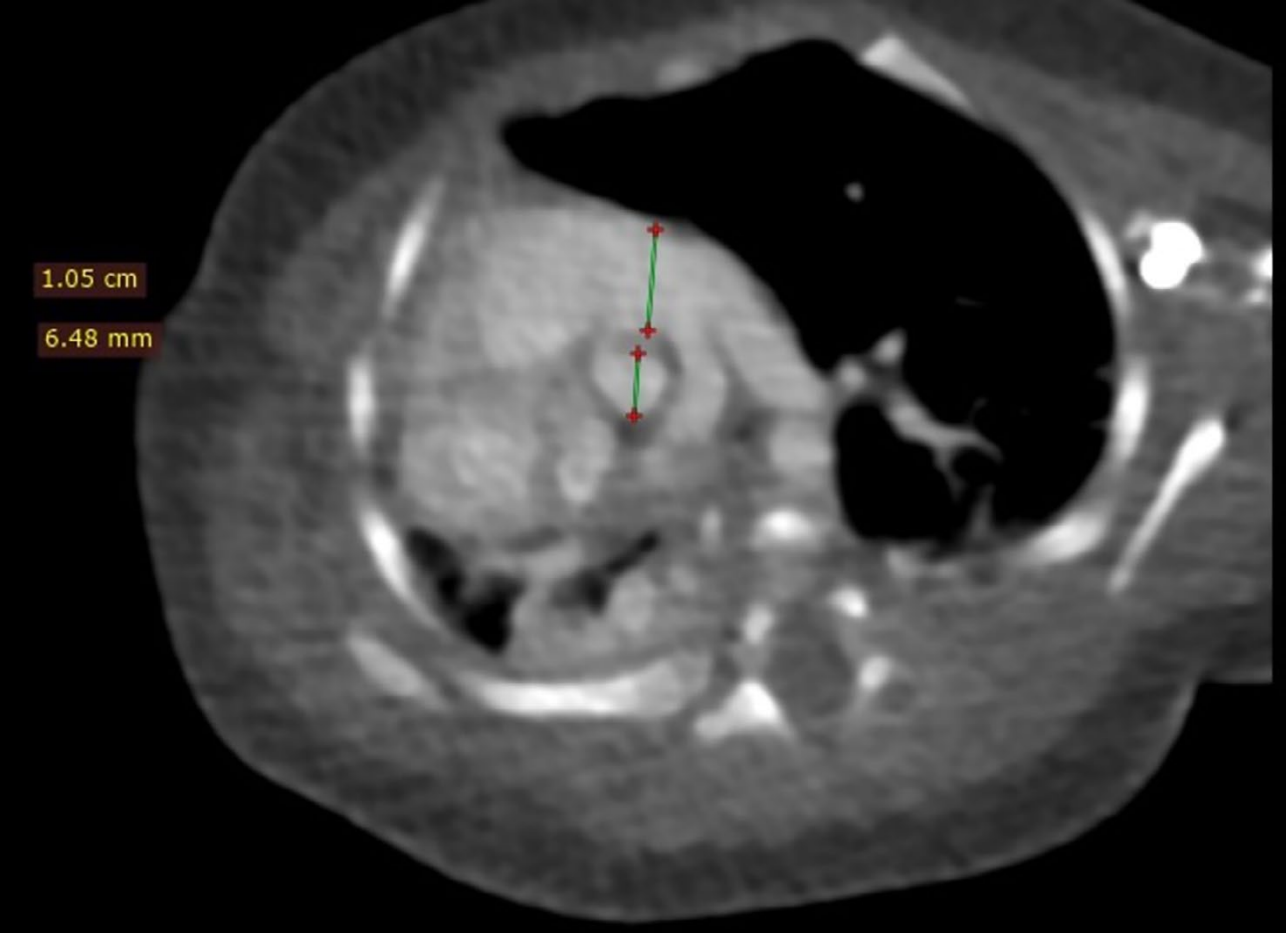
A post IV contrast CT of the chest, an axial slice at the level of the great vessels, soft tissue window which is demonstrating a visibly enlarged main pulmonary trunk with a pulmonary trunk to aortic ratio of more than 1.6. The right pulmonary artery is also slightly smaller than the left pulmonary artery. With the exception of right‐sided rib crowding, there were no overlying bony structural defects noted.

Additionally, a close examination of the great vessels (Figure 3) illustrated an enlarged main pulmonary trunk, with a pulmonary‐to‐aortic diameter ratio exceeding 1.6. The right pulmonary artery was noted to be slightly smaller than the left, and while right‐sided rib crowding was evident, no significant bony structural abnormalities were identified. With these findings, pulmonary hypertension was suspected due to radiological findings of disproportionate aortic‐to‐main pulmonary artery ratio; no echocardiography was done on the patient.

No other structural abnormalities were detected in the scan; however, hepatomegaly was observed during the evaluation of other organs. No other comorbidities were identified since the renal function tests were normal and the CTA did not show any anomalies of the renal parenchyma. The renal function tests were within normal ranges.

## 3 Differential Diagnosis

In neonates presenting with severe respiratory distress and cyanosis, it is essential to differentiate congenital pulmonary hypoplasia from other conditions that may mimic its presentation. The differential diagnosis included congenital diaphragmatic hernia (CDH), which can present with similar respiratory symptoms due to impaired lung development. CDH can often be ruled out with imaging that shows diaphragm integrity, as was done in this case. Other differential considerations included congenital lobar emphysema, which may cause respiratory distress and hyperinflation but typically presents with lobar over inflation rather than hypoplasia. Pulmonary sequestration, another anomaly with aberrant lung tissue development, can also cause respiratory symptoms but is typically associated with an anomalous blood supply. In this case, imaging showed no such aberrant vasculature. Congenital heart disease was also considered in neonates with persistent hypoxemia; however, the absence of cardiac structural abnormalities on imaging helped narrow down the diagnosis to unilateral pulmonary hypoplasia. Respiratory distress syndrome, meconium aspiration syndrome, congenital pneumonia were also considered as possible differentials.

## 4 Conclusion and Results (Outcome and Follow‐Up)

This case of unilateral congenital pulmonary hypoplasia in a three‐week‐old neonate highlighted the importance of prompt and accurate diagnosis in neonates presenting with severe respiratory distress. CTA was crucial in confirming the diagnosis by identifying significant hypoplasia of the right lung, compensatory hyperinflation of the left lung, and pulmonary hypertension. Despite ventilator support in the neonatal intensive care unit, the neonate's respiratory function remained compromised, and no surgical intervention was planned at this stage due to the poor prognosis. Follow‐up in this case included continued supportive care to manage respiratory distress and routine monitoring for any potential complications related to pulmonary hypertension and compromised lung function. The patient passed a few weeks later while still in NICU. The cause of death was not ascertained since the parents declined an autopsy. This case shows the critical role of advanced imaging in guiding management and setting realistic expectations for outcomes in neonates with congenital pulmonary hypoplasia.

## 5 Discussion

Congenital lung hypoplasia is a rare entity arising from underdevelopment of one or both lungs during fetal growth. Unilateral congenital pulmonary hypoplasia, as in our case, is an even rarer occurrence that may occur in isolation, or with other syndromes [3, 6, 8]. The pathophysiology of pulmonary hypoplasia is still debatable but some literature suggests it is due to lung insult in the fetal stage of life [3] agreeing with our case who was born with the condition. We cannot rule out with certainty that the lung could have been underdeveloped due to some other cause, with the resulting abnormal vascular resistance/pressure explaining the dilated left pulmonary artery. In essence, lung underdevelopment can result in vascular underdevelopment and vice versa. With an incidence of 9–11 per 10,000 births, there is no reported sex predilection and frequency occurs in males and females alike [2, 4, 7].

The causes of pulmonary hypoplasia in this neonate could not readily be ascertained but we postulate that the baby could have had a vascular insult to the pulmonary arteries affecting the right more than the left with resulting lung injury and this can be explained by the discrepancy in sizes of the right and left pulmonary arteries. Studies have in fact reported vascular compromise in fetal life since bronchopulmonary segments develop relative to each other [6, 9, 10]. Other causes of pulmonary hypoplasia could not be confirmed like genetic causes due to the lack of infrastructure for conducting tests in the country, prior maternal‐fetal infection with cytomegalovirus, or chromosomal anomalies since the tests were not done; we however ruled out congenital diaphragmatic hernia, abdominal wall defects, skeletal dysplasia and intrathoracic masses by the CTA which are noted by some literature [2, 3, 10].

Clinical presentation of congenital lung hypoplasia is nonspecific but a high index of suspicion should be held when the neonate develops respiratory distress immediately after birth that does not improve with other interventions such as ventilation, surfactant administration etc. [1, 4, 7]. Other signs in the case discussed here included suboptimal oxygen saturation, an acidotic picture of the arterial blood gases and absence of fever suggestive of a non‐infectious process which have all been reported in similar studies [1, 3, 6].

CTA has emerged as a valuable imaging modality in the evaluation of congenital lung hypoplasia due to its high‐resolution imaging capabilities and ability to provide detailed information about lung structures and vascular anatomy, but judicious use should be applied on a case‐by‐case basis [1, 5, 6]. In the case of our 3‐week‐old neonate, CTA was employed as part of the diagnostic workup during his NICU stay to confirm the presence and extent of lung hypoplasia. CTA was selected primarily because the imaging unit is located adjacent to the NICU, allowing for quicker and safer imaging of the critically ill neonate. The lower cost of CTA compared to MRA was a secondary consideration. However, in this particular case, the patient's family would not have been able to afford MRA regardless. This highlights the need to improve the integration of MRI services into NICUs, especially in facilities like ours, where both access and affordability remain significant barriers. The ALARA principle was used; as low as reasonably achievable for informative images to be obtained and since it was not yet determined what the cause of distress was, both the chest and abdomen were included in the region of interest. The procedure allowed for a comprehensive three‐dimensional visualization of the neonate's lung parenchyma, bronchial tree, and pulmonary vasculature such as pulmonary artery dilatation, stenosis or hypoplasia enabling a more precise evaluation compared to conventional imaging techniques like chest X‐rays. It is worth noting that the clinical benefits of CTA in confirming the diagnosis and guiding management decisions often outweigh the risks when performed judiciously.

The findings from CTA in this case were a reduced lung volume of the right lung with ipsilateral shift of mediastinal structures, fibrotic changes and consolidation, an enlarged main pulmonary trunk with an intact diaphragm, thoracic cage arrangement which is a pointer towards primary congenital unilateral pulmonary hypoplasia complicated by consolidative fibrosis and pulmonary hypertension. These findings are agreeable to most existing literature regarding congenital pulmonary hyperplasia as well as the potential complications [3, 5, 6, 7, 11].

The prognosis of the neonate was poor as he did not improve despite the NICU interventions and no surgical plan was implemented. The child died a few weeks later while still in the NICU. We note the general poor prognosis and high mortality rate associated with this condition which tallies with current available literature [3, 4, 8].

## Author Contributions

**Bernadette Pedun:** conceptualization, data curation, formal analysis, investigation, methodology, project administration, resources, software, supervision, validation, visualization, writing – original draft, writing – review and editing. **Ivaan Pitua:** formal analysis, methodology, software, writing – original draft, writing – review and editing. **Samuel Bugeza:** data curation, formal analysis, investigation, methodology, writing – original draft, writing – review and editing. **Anthony Oriekot:** methodology, validation, writing – original draft, writing – review and editing. **Felix Bongomin:** formal analysis, methodology, supervision, writing – original draft, writing – review and editing.

## Ethics Statement

No institutional approval was required to publish the case details.

## Consent

Written informed consent for publication of this case report and accompanying images was obtained from the patient's parent.

## Conflicts of Interest

The authors declare no conflicts of interest.

## Data Availability

All relevant data and materials are availed within the text.

## References

[1] M. E. Abrams , V. L. Ackerman , and W. A. Engle , “Primary Unilateral Pulmonary Hypoplasia: Neonate Through Early Childhood—Case Report, Radiographic Diagnosis and Review of the Literature,” Journal of Perinatology 24, no. 10 (2004): 667–670. 15454946 10.1038/sj.jp.7211156

[2] N. Cregg and W. Casey , “Primary Congenital Pulmonary Hypoplasia—Genetic Component to Aetiology,” Pediatric Anesthesia 7, no. 4 (1997): 329–333. 9243692 10.1046/j.1460-9592.1997.d01-81.x

[3] K. Fujioka , I. Morioka , K. Nishida , et al., “Pulmonary Hypoplasia Caused by Fetal Ascites in Congenital Cytomegalovirus Infection Despite Fetal Therapy,” Frontiers in Pediatrics 5 (2017): 241. 29164089 10.3389/fped.2017.00241 PMC5681744

[4] H. Jui‐Sheng , L. Yu‐Sheng , L. Chin‐Hsuan , et al., “Primary Congenital Pulmonary Hypoplasia of a Neonate,” Journal of the Chinese Medical Association 75, no. 2 (2012): 87–90. 22340744 10.1016/j.jcma.2011.12.004

[5] P. Kosiński and M. Wielgoś , “Congenital Diaphragmatic Hernia: Pathogenesis, Prenatal Diagnosis and Management — Literature Review,” Ginekologia Polska 88, no. 1 (2017): 24–30. 28157247 10.5603/GP.a2017.0005

[6] N. O. Elhassan , C. Sproles , R. Sachdeva , S. T. Bhutta , and J. S. Szabo , “A Neonate With Left Pulmonary Artery Thrombosis and Left Lung Hypoplasia: a Case Report,” Journal of Medical Case Reports 4 (2010): 284. 20731840 10.1186/1752-1947-4-284 PMC2933637

[7] S. Menahem , A. Sehgal , and D. F. Wurzel , “Persistent Tachypnoea in Early Infancy: A Clinical Perspective,” Children (Basel) 10, no. 5 (2023): 789. 37238337 10.3390/children10050789 PMC10216969

[8] K. R. Ellsworth , M. A. Ellsworth , A. L. Weaver , K. C. Mara , R. H. Clark , and W. A. Carey , “Association of Early Inhaled Nitric Oxide With the Survival of Preterm Neonates With Pulmonary Hypoplasia,” JAMA Pediatrics 172, no. 7 (2018): e180761. 29800952 10.1001/jamapediatrics.2018.0761 PMC6137510

[9] I. Caldeira , H. Fernandes‐Silva , D. Machado‐Costa , J. Correia‐Pinto , and R. S. Moura , “Developmental Pathways Underlying Lung Development and Congenital Lung Disorders,” Cells 10, no. 11 (2021): 2987. 34831210 10.3390/cells10112987 PMC8616556

[10] D. T. Swarr , W. H. Peranteau , J. Pogoriler , et al., “Novel Molecular and Phenotypic Insights Into Congenital Lung Malformations,” American Journal of Respiratory and Critical Care Medicine 197, no. 10 (2018): 1328–1339. 29328793 10.1164/rccm.201706-1243OC PMC5955056

[11] V. Sharma , S. Berkelhamer , and S. Lakshminrusimha , “Persistent Pulmonary Hypertension of the Newborn,” Maternal Health, Neonatology and Perinatology 1, no. 1 (2015): 14. 27057331 10.1186/s40748-015-0015-4 PMC4823682

